# Mitogenomic diversity and phylogenetic characterization of *Aedes albopictus* (Diptera: Culicidae) populations from the Black Sea region of Türkiye

**DOI:** 10.1093/jme/tjag109

**Published:** 2026-07-03

**Authors:** Samba Deguene Diop, Alparslan Yildirim, Batuhan Askim Arslanhan, Ahsen Meliha Toroslu, Gokmen Zafer Pekmezci, Simge Sahin, Denis Alina Kizgin, Saffet Teber, Osman Ibis, Filiz Gunay, Nathan Burkett-Cadena, Lindsay P Campbell, Abdullah Inci, Barry Alto

**Affiliations:** Faculty of Veterinary Medicine, Parasitology Department, Erciyes University, Kayseri, Türkiye; Health Science Institute, Erciyes University, Erciyes University, Kayseri, Türkiye; Faculty of Veterinary Medicine, Parasitology Department, Erciyes University, Kayseri, Türkiye; Faculty of Veterinary Medicine, Parasitology Department, Erciyes University, Kayseri, Türkiye; Health Science Institute, Erciyes University, Erciyes University, Kayseri, Türkiye; Faculty of Veterinary Medicine, Department of Parasitology, Ondokuz Mayis University, Samsun, Türkiye; Faculty of Veterinary Medicine, Department of Preclinical Sciences, Ondokuz Mayis University, Samsun, Türkiye; Faculty of Veterinary Medicine, Parasitology Department, Erciyes University, Kayseri, Türkiye; Health Science Institute, Erciyes University, Erciyes University, Kayseri, Türkiye; Faculty of Veterinary Medicine, Parasitology Department, Erciyes University, Kayseri, Türkiye; Health Science Institute, Erciyes University, Erciyes University, Kayseri, Türkiye; Faculty of Agriculture, Department of Agricultural Biotechnology, Erciyes University, Kayseri, Türkiye; Faculty of Agriculture, Department of Agricultural Biotechnology, Erciyes University, Kayseri, Türkiye; Florida Medical Entomology Laboratory, University of Florida, IFAS, Vero Beach, FL, USA; Biology Department, Ecology Section, Hacettepe University, Ankara, Türkiye; Florida Medical Entomology Laboratory, University of Florida, IFAS, Vero Beach, FL, USA; Department of Entomology and Nematology, IFAS, University of Florida, Gainesville, FL, USA; Florida Medical Entomology Laboratory, University of Florida, IFAS, Vero Beach, FL, USA; Department of Entomology and Nematology, IFAS, University of Florida, Gainesville, FL, USA; Faculty of Veterinary Medicine, Parasitology Department, Erciyes University, Kayseri, Türkiye; Florida Medical Entomology Laboratory, University of Florida, IFAS, Vero Beach, FL, USA; Department of Entomology and Nematology, IFAS, University of Florida, Gainesville, FL, USA

**Keywords:** mitogenome, phylogeography, invasive mosquito, maternal lineage, population structure

## Abstract

*Aedes albopictus* (Skuse, 1894), the Asian tiger mosquito, is one of the most invasive mosquito species worldwide and an important vector of several arboviruses of public health concern. Although its distribution in Türkiye has expanded rapidly since its first detection, the mitogenomic diversity and phylogeographic relationships of Turkish populations remain poorly understood. Here, we provide the first mitogenome-based characterization of *Ae. albopictus* populations from the Black Sea region of Türkiye within a global comparative framework. Complete mitochondrial genomes were generated from 20 specimens collected along the Turkish Black Sea coast and analyzed together with 75 reference mitogenomes retrieved from GenBank. The Turkish mitogenomes ranged from 15.8 to 17.1 kb and exhibited the typical *Ae. albopictus* mitochondrial organization, including 13 protein-coding genes, 22 transfer RNAs, 2 ribosomal RNAs, and a control region. Gene order and orientation were conserved across all Turkish samples, and most size variation was confined to the control region. The mitogenomes were strongly AT-rich, and codon usage showed a marked bias toward A/U-ending codons. Phylogenetic analyses using concatenated sequences of all 13 protein-coding genes and both ribosomal RNA genes showed that Turkish samples were not monophyletic; rather, they were distributed among multiple subclades within the broad A1a complex. The results indicate high mitogenomic diversity and a mosaic phylogeographic structure, consistent with multiple maternal lineages contributing to *Ae. albopictus* populations in Türkiye’s Black Sea region. The phylogenetic signature supports a scenario of multiple introductions of *Ae. albopictus* rather than spread from a single founding population.

## Introduction

*Aedes albopictus* (Skuse, 1894), commonly known as the Asian tiger mosquito, is one of the most invasive mosquito species worldwide and an important public health concern (Benedict et al. 2007, Paupy et al. 2009, Bonizzoni et al. 2013). Native to the tropical forests of Southeast Asia, this species has successfully colonized every continent except Antarctica during the last 50 years (Rai 1991, Bonizzoni et al. 2013). The rapid global expansion of this anthropogenic species has been attributed to its ecological plasticity and its physiological and behavioral traits.

The concept of “anthropogenically induced adaptation to invade” (AIAI) proposes that adaptations or traits present in a species’ native range can predispose it to succeed in newly invaded environments (Hufbauer et al. 2012), a framework applied to *Ae. albopictus* to explain its notable invasive success (Juliano and Lounibos 2016). In its native Asian range, *Ae. albopictus* is an ecotonal species occurring primarily along forest fringes (Lounibos et al. 2001), a preference mirrored in its invasive ranges and likely contributing to its global spread (Juliano and Lounibos 2016).

*Aedes albopictus* is also associated with human-dominated habitats, where females exhibit flexible host-feeding patterns (Hawley 1988, Garamszegi 2024), lay desiccation-resistant and diapausing eggs, and utilize container habitats for immature development. In addition, *Ae. albopictus* often outcompetes other mosquito species during interspecific larval competition, particularly in low-resource environments (Livdahl and Willey 1991, Juliano 1998, Costanzo et al. 2005, Armistead et al. 2008, Juliano 2010, Costanzo et al. 2011, Bonizzoni et al. 2013, Alto et al. 2025). Finally, asymmetric reproductive interference (satyrization) has been proposed as a mechanism facilitating the displacement of *Ae. aegypti*. Interspecific mating occurs where their ranges overlap, resulting in the sterilization of *Ae. aegypti* females, but not *Ae. albopictus*, due to accessory gland substances transferred by *Ae. albopictus* males (Tripet et al. 2011, Bargielowski and Lounibos 2016).

The worldwide spread of *Ae. albopictus* has been facilitated by the unintentional transport of eggs in artificial and natural containers, especially through the used tire trade and Lucky bamboo trade, as well as by passive dispersal via ground vehicles associated with its anthropophilic behavior (Lounibos 2002, Bonizzoni et al. 2013). As a competent vector of several arboviruses, including dengue, Zika, chikungunya, and yellow fever viruses, *Ae. albopictus* poses a substantial threat to global public health (Gratz 2004, Paupy et al. 2009). Its recent expansion has also raised concern about future mosquito-borne disease outbreaks in newly invaded urban and peri-urban areas (Bonizzoni et al. 2013).

In Türkiye, *Ae. albopictus* was first detected in 2011 in the Thrace region of northwestern Türkiye (Oter et al. 2013). Subsequent surveillance revealed its spread to other parts of the country, including the Black Sea coast (Akiner et al. 2016, Demirci et al. 2021). By 2019, the species had been reported from five geographical regions of Türkiye, namely the Black Sea, Central Anatolia, Marmara, Mediterranean, and Aegean regions (Demirci et al. 2021). Given its rapid spread and increasing public health relevance, understanding the genetic structure and phylogeographic patterns of *Ae. albopictus* populations in Türkiye is important.

Mitochondrial DNA (mtDNA) has been widely used to investigate genetic relationships and phylogeographic structure in *Ae. albopictus* populations (Usmani-Brown et al. 2009, Battaglia et al. 2016). The mitochondrial genome is a small circular molecule, typically 15–20 kb in length, that contains 37 genes: 13 protein-coding genes, 22 transfer RNA genes, and 2 ribosomal RNA genes. Because of its maternal inheritance, lack of recombination, and relatively rapid evolutionary rate, mtDNA is well suited for reconstructing phylogenetic and phylogeographic relationships within species (Usmani-Brown et al. 2009, Battaglia et al. 2016). Previous studies identified several mitochondrial haplogroups in *Ae. albopictus* worldwide. Battaglia et al. (2016) defined 5 major haplogroups (A1a1, A1a2, A1b, A2, and A3), of which 3 (A1a1, A1a2, and A1b) were likely involved in the recent global spread of the species. Subsequent work has further refined the distribution of these haplogroups and clarified their potential geographic origins (Battaglia et al. 2022).

In the present study, we analyzed, for the first time, mitogenome variation in 20 *Ae. albopictus* specimens collected from the Black Sea region of Türkiye in the context of available global data. Our objectives were to characterize mitogenomic diversity in Turkish populations, assess their phylogenetic placement within the currently recognized mitochondrial haplogroup framework, and explore possible maternal lineage affinities. This study contributes to a better understanding of the population structure of *Ae. albopictus* in a previously understudied region and provides a mitogenomic perspective on the diversity of Turkish populations within the global invasive range of the species.

## Materials and Methods

### Mosquito Collection and Identification

**Fig. 1.**
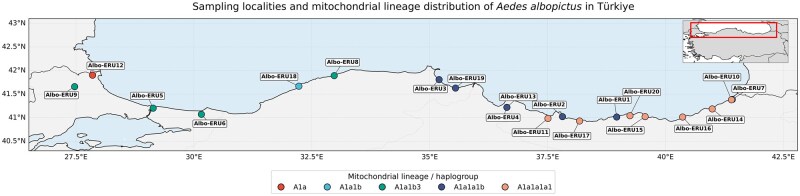
Sampling localities and mitochondrial lineage/haplogroup distribution of Turkish *Ae. albopictus* isolates along the Black Sea coast of Türkiye. Each point represents one isolate and is color-coded according to its mitochondrial lineage/haplogroup assignment. The distribution of lineages indicates a mosaic-like geographic pattern rather than a simple west-to-east gradient.

Eggs, larvae, and adults of *Ae. albopictus* (Skuse, 1894) were collected along the Turkish Black Sea coast as part of the project “Enhancing arbovirus surveillance and risk management in the public health systems of Georgia, Türkiye, and Ukraine” (Grant/Award No. HDTRA12210015; Fig. 1). Ovitrap sampling was performed at collection sites from May to September 2024 using black plastic cups containing grass infusion (Sant’ana et al. 2006) as an attractant and a cardboard paddle as an oviposition substrate. Ovitraps were deployed in the field for six days, after which the cardboard paddles and fluid contents of the container were collected and transferred into plastic containers. Subsequently, eggs and larvae were transported to the insectary of the Faculty of Veterinary Medicine at Erciyes University, where immature stages were reared to adulthood under controlled conditions of 26 ± 2 °C, 55–65% relative humidity, and a photoperiod of 18:6 (L:D) h. One emerged adult from each selected location was used for downstream analyses. Specimens were morphologically identified using a standard taxonomic key (Günay et al. 2020). Individuals identified as *Ae. albopictus* were used for genomic DNA extraction.

### DNA Extraction, Library Preparation, and Sequencing

Genomic DNA was extracted individually from each mosquito specimen using the PureLink™ Genomic DNA Mini Kit (Invitrogen, Thermo Fisher Scientific, Waltham, MA, USA) following the manufacturer’s instructions. The DNA concentration of each mosquito sample was determined using the Qubit DNA BR assay and standardized to a concentration of 0.2 ng/μl for library preparation. A separate genomic library was prepared for each mosquito specimen. DNA was fragmented and labeled with adapter sequences (i7 and i5) using the Nextera XT DNA library preparation kit (Illumina) and purified with Agencourt AMPure XP beads (Beckman Coulter), following the manufacturer’s recommendations. The quality of the genomic libraries was assessed by quantifying fragments using a Qubit 4.0 fluorometer (Life Technologies) and verifying fragment size using a Bioanalyzer 2100 (Agilent Technologies) with the High Sensitivity DNA Analysis kit, according to the manufacturer’s protocols. Finally, the sequencing of the products obtained from the genomic libraries was performed using the NextSeq 500 System (Illumina) platform, using the NextSeq 500/550 High Output kit v2.5 (Illumina), in a run of 300 cycles (2 × 150), following the protocol standards established by the manufacturer. Details of the *Ae. albopictus* isolates, including sampling location, geographic coordinates, collection date, numbers of trimmed and quality-filtered reads, assembly information, and mitogenome length, are provided in Supplementary Table S1.

### Mitogenome Assembly and Annotation

Raw sequence quality for the 20 *Ae. albopictus* samples was assessed using FastQC (Andrews 2010). Reads shorter than 50 bp were removed, low-quality bases at read ends (*Q* score <25) were trimmed, and adapter sequences were removed using the BBDuk plugin in Geneious Prime version 2025.0.3's (https://www.geneious.com). For each sample, filtered paired-end reads (approximately 400,000 read pairs per sample) were mapped individually to the *Ae. albopictus* reference mitochondrial genome (GenBank accession NC_006817) in Geneious Prime using the Map to Reference function and the Highest Sensitivity/Medium Sensitivity setting with up to 25 iterations. To verify completeness, de novo assembly was also performed for each sample using GetOrganelle v1.7.7.0 (Jin et al. 2020). De novo contigs were compared with reference-based assemblies to confirm sequence consistency. All 20 mitogenome assemblies were annotated using MITOS2 (Bernt et al. 2013) under the invertebrate mitochondrial genetic code, and gene boundaries were manually checked. The final annotations included 13 protein-coding genes, 22 transfer RNA genes, 2 ribosomal RNA genes, and a control region. All 20 assembled mitochondrial genomes were deposited in GenBank under accession numbers PZ201005–PZ201024.

### Mitogenome Characterization

Annotated mitogenomes were analyzed using a custom Python pipeline with Biopython for sequence and annotation extraction. For each Turkish isolate, the 13 protein-encoding genes, 22 transfer RNAs, and 2 ribosomal RNAs were extracted from GenBank feature tables. Gene strand distribution, overlaps, intergenic regions, and control region length were also recorded. Quantitative variables were summarized as mean ± standard deviation and observed range, while invariant structural traits were reported as consensus states. For the 13 mitochondrial protein genes, lengths, start codons, stop codons, and strand orientation were compared between isolates. The nucleotide composition of the complete mitogenome and its main partitions was assessed by calculating the proportions of A, T, G, and C, as well as the A+T, G+C, AT-skew, and GC-skew indices. The relative use of synonymous codons (RSCU) was estimated from the concatenated set of 13 PCGs according to the mitochondrial genetic code of invertebrates.

### Phylogenetic Analyses

Complete and partial mitochondrial genome records of *Ae. albopictus* were retrieved from NCBI GenBank and used as the reference dataset for phylogenetic reconstruction (Benson et al. 2018). The global reference mitogenomes and newly generated Turkish isolates included in the phylogenetic dataset are listed in Table 1. Except for this initial download step, all subsequent analyses were carried out using custom scripts in Python, mainly with Bio.Entrez, Bio.SeqIO, and Bio.Phylo from Biopython, together with pandas and subprocess. The phylogenetic matrix was based on the 13 canonical mitochondrial protein-coding genes (PCGs) plus the 12S rRNA and 16S rRNA loci, consistent with the typical mitochondrial genome organization of mosquitoes (Zhang et al. 2016). Multiple sequence alignments were generated independently for each locus using MAFFT v7 with the—auto option (Katoh and Standley 2013). The aligned loci were then concatenated into a single supermatrix while preserving gene boundaries for partitioned phylogenetic inference. Phylogenetic reconstruction was performed under a partitioned maximum-likelihood (ML) framework using RAxML-NG (Kozlov et al. 2019), with each of the 15 loci treated as an independent partition under a GTR+G model scheme (Yang 1994). Branch support was assessed using 1,000 nonparametric bootstrap replicates (Felsenstein 1985), and the final ML tree was exported in Newick format for annotation and visualization. Turkish samples were sequentially renamed Albo-ERU1 to Albo-ERU20. Final tree-tip labels were formatted as GenBank_ID | original isolate name | country | haplogroup. One metadata entry corresponding to NC_006817 was excluded from the final relabeling table because it was not part of the analyzed 95-isolate tree.

**Table 1.** Global reference mitogenomes and newly generated Turkish *Aedes albopictus* isolates used in this study

Sequence ID	Original name	Country, place of collection	Haplogroup	GenBank ID
**1**	Rim1	Italy, Rimini	A1a1a1a1	KX383916
**2**	J-To1	Japan, Tokyo	A1a1a1a1	MH587188
**3**	J-Ka1	Japan, Kanagawa	A1a1a1a1	MH587189
**4**	VB2	United States, Florida, Vero Beach	A1a1a1a1	MH587190
**5**	LA1	United States, California, Los Angeles	A1a1a1a1a	MH587191
**6**	LA2	United States, California, Los Angeles	A1a1a1a1a	MH587192
**7**	LA3	United States, California, Los Angeles	A1a1a1a1a	MH587193
**8**	LA8	United States, California, Los Angeles	A1a1a1a1a	MH587194
**9**	LA11	United States, California, Los Angeles	A1a1a1a1a	MH587195
**10**	LA4	United States, California, Los Angeles	A1a1a1a1a	MH587196
**11**	VB3	United States, Florida, Vero Beach	A1a1a1a1	MH587197
**12**	VB4	United States, Florida, Vero Beach	A1a1a1a1	MH587198
**13**	Vir1	United States, Virginia	A1a1a1a1b1	KX383917
**14**	Rc1	Italy, Reggio Calabria	A1a1a1a1b1	KX383918
**15**	Vir2	United States, Virginia	A1a1a1a1b1	KX383919
**16**	Ces1	Italy, Cesena	A1a1a1a1b1	KX383920
**17**	CRM4	Italy, Crema	A1a1a1a1b1	MH587217
**18**	Cas1	Italy, Cassino	A1a1a1a1b	KX383921
**19**	Pav3	Italy, Pavia	A1a1a1a1b	KX383922
**20**	PoMo2600	Portugal, Oporto	A1a1a1a1b	MN513353
**21**	PoMo2601	Portugal, Oporto	A1a1a1a1b	MN513354
**22**	PoMo2604	Portugal, Oporto	A1a1a1a1b	MN513356
**23**	PoMo2608	Portugal, Oporto	A1a1a1a1b	MN513358
**24**	Mex1	Mexico, Tapachula	A1a1a1a1b	MH587199
**25**	J-Fu1	Japan, Fukushima	A1a1a1a1b	MH587200
**26**	J-Wa2	Japan, Wakayama	A1a1a1a	MH587201
**27**	J-Wa3	Japan, Wakayama	A1a1a1b	MH587202
**28**	J-Wa1	Japan, Wakayama	A1a1a1b	KX809765
**29**	Cu1	France, Cuers, Var, PACA	A1a1a1b	MH587203
**30**	PoMo2728	Portugal, Algarve	A1a1a1b	MN513362
**31**	PoMoF636	Portugal, Algarve	A1a1a1b	MN513368
**32**	Ces2	Italy, Cesena	A1a1a	KX383923
**33**	CC1	Italy, Perugia	A1a1b	MH587204
**34**	Cu4	France, Cuers, Var, PACA	A1a1b	MH587219
**35**	PoMo2599	Portugal, Algarve	A1a1b	MN513352
**36**	PoMo2711	Portugal, Algarve	A1a1b	MN513361
**37**	PoMo2708	Portugal, Algarve	A1a1b	MN513359
**38**	Cu2	France, Cuers, Var, PACA	A1a1b1a	MH587205
**39**	Rim4	Italy, Rimini	A1a1b1a	KX383929
**40**	Tir1	Albania, Tirana	A1a1b1a	KX383930
**41**	Tir2	Albania, Tirana	A1a1b1a	KX383931
**42**	—	China, Jiangsu, Nanjing	A1a1b1a	KR068634
**43**	Ath2	Greece, Athens	A1a1b1	KX383932
**44**	PoMo2607	Portugal, Oporto	A1a1b1	MN513357
**45**	Pav4	Italy, Pavia	A1a1b1	KX383933
**46**	Co1	Italy, Reggio Emilia	A1a1b1	MH587206
**47**	Fo2	China, Foshan	A1a1b2a	KX383934
**48**	Fo4	China, Foshan	A1a1b2a	MH587207
**49**	Fo1	China, Foshan	A1a1b2a	MH587220
**50**	Fo5	China, Foshan	A1a1b2a	MH587221
**51**	FPA1	China, Foshan	A1a1b2a	MH587222
**52**	FPA2	China, Foshan	A1a1b2a	MH587223
**53**	FPA3	China, Foshan	A1a1b2a	MH587224
**54**	PoMo2602	Portugal, Oporto	A1a1b2	MN513355
**55**	PoMoF505	Portugal, Oporto	A1a1b2	MN513364
56	Cu5	France, Cuers, Var, PACA	A1a	MH587218
**57**	PoMoF506	Portugal, Algarve	A1a	MN513365
**58**	PoMoF607	Portugal, Algarve	A1a	MN513366
**59**	RdJ1	Brazil, Rio de Janeiro	A1b1	MH587208
**60**	RdJ2	Brazil, Rio de Janeiro	A1b1	MH587209
**61**	RdJ4	Brazil, Rio de Janeiro	A1b1	MH587211
**62**	RdJ3	Brazil, Rio de Janeiro	A1b1	MH587210
**63**	Lam2	Thailand, Lampang, Hang Chat	A1b2a	KX383925
**64**	Ban7	Thailand, Uthai Thani, Ban Rai	A1b2a	KX383926
**65**	Ath1	Greece, Athens	A1b2a1	KX383927
**66**	Cam2	Cameroon	A1b2a1	MH587212
**67**	Cam3	Cameroon	A1b2a1	MH587213
**68**	VB1	United States, Florida, Vero Beach	A1b2a1	MH587214
**69**	Cam1	Cameroon	A1b2b	MH587215
**70**	Cam4	Cameroon	A1b2b	MH587216
**71**	Los1	Philippines, Laguna, Los Baños	A2a	KX383935
**72**	Los2	Philippines, Laguna, Los Baños	A2a	KX809761
**73**	Los3	Philippines, Laguna, Los Baños	A2a	KX809762
**74**	Los5	Philippines, Laguna, Los Baños	A2a	KX809764
**75**	Los4	Philippines, Laguna, Los Baños	A2	KX809763
**76**	…	Taiwan, Taipei	A3	NC006817
**77**	Albo-ERU1	Türkiye	A1a1a1b	PZ201005
**78**	Albo-ERU2	Türkiye	A1a1a1b	PZ201006
**79**	Albo-ERU3	Türkiye	A1a1a1b	PZ201007
**80**	Albo-ERU4	Türkiye	A1a1a1b	PZ201008
**81**	Albo-ERU5	Türkiye	A1a1b3	PZ201009
**82**	Albo-ERU6	Türkiye	A1a1b3	PZ201010
**83**	Albo-ERU7	Türkiye	A1a1b	PZ201011
**84**	Albo-ERU8	Türkiye	A1a1b3	PZ201012
**85**	Albo-ERU9	Türkiye	A1a1b3	PZ201013
**86**	Albo-ERU10	Türkiye	A1a1a1a1	PZ201014
**87**	Albo-ERU11	Türkiye	A1a1a1a1	PZ201015
**88**	Albo-ERU12	Türkiye	A1a	PZ201016
**89**	Albo-ERU13	Türkiye	A1a1a1b	PZ201017
**90**	Albo-ERU14	Türkiye	A1a1a1a1	PZ201018
**91**	Albo-ERU15	Türkiye	A1a1a1a1	PZ201019
**92**	Albo-ERU16	Türkiye	A1a1a1a1	PZ201020
**93**	Albo-ERU17	Türkiye	A1a1a1a1	PZ201021
**94**	Albo-ERU18	Türkiye	A1a1b	PZ201022
**95**	Albo-ERU19	Türkiye	A1a1a1b	PZ201023
**96**	Albo-ERU20	Türkiye	A1a1a1a1	PZ201024

## Results

### Mitogenome Assembly and General Features

**Fig. 2.**
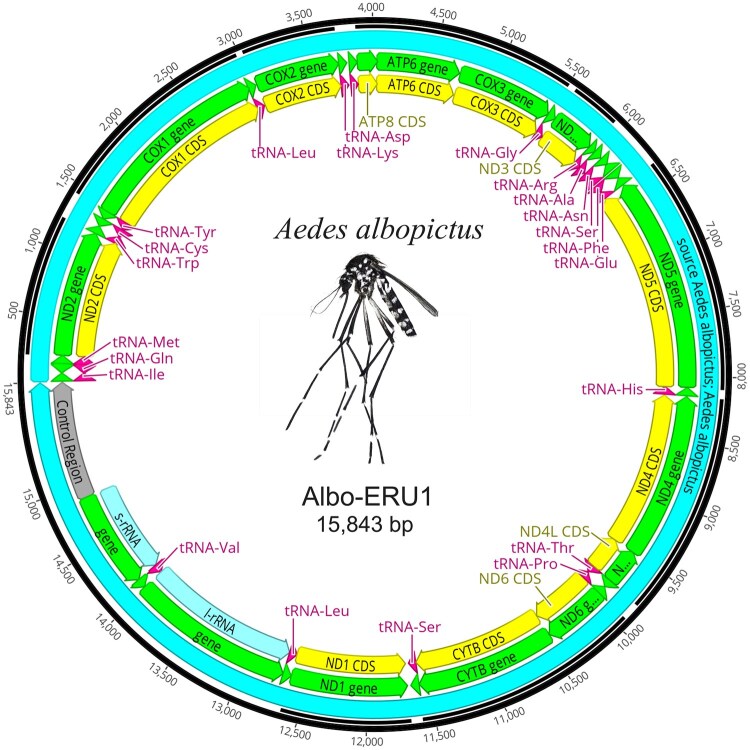
Representative mitochondrial genome map of *Ae. albopictus* from Türkiye.

Complete mitochondrial genomes were obtained for all 20 Turkish *Ae. albopictus* isolates. Mitogenome length ranged from 15.8 to 17.1 kb. All assemblies displayed the typical *Ae. albopictus* mitochondrial organization, comprising 13 protein-coding genes, 22 transfer RNAs, 2 ribosomal RNAs, and a control region. Gene order and orientation were identical across all samples and matched the reference mitogenome (GenBank accession NC_006817). No gene rearrangements were detected. The orientation of ND1, ND4, ND4L, and ND5 was conserved on the reverse strand, whereas ATP6, ATP8, COX1–COX3, CYTB, ND2, ND3, and ND6 were consistently located on the forward strand (Fig. 2). The coding regions were highly conserved among Turkish isolates, with no variation in the lengths of most protein-coding genes. For example, COX1 was 1,539 bp, ND5 was 1,743 bp, ND4 was 1,344 bp, CYTB was 1,137 bp, ATP6 was 681 bp, and ATP8 was 162 bp in all isolates. The 12S rRNA gene was constant in length (797 bp), whereas the 16S rRNA gene varied only slightly (1,336–1,337 bp). Transfer RNA genes showed little size variation (64–72 bp). Most length variation among the 20 mitogenomes was confined to the control region, which ranged from 948 to 2,184 bp (Table 2).

**Table 2.** Consensus gene organization and annotation features of the 20 Turkish *Ae. albopictus* mitogenomes

Gene	Strand	Consensus length (bp)	Observed range (bp)	Start codon	Stop codon
**trnI**	J-strand	69	69–69	…	…
**trnQ**	N-strand	69	69–69	…	…
**trnM**	J-strand	69	69–69	…	…
**ND2**	J-strand	1026	1026–1026	ATT	TAA
**trnW**	J-strand	69	69–69	…	…
**trnC**	N-strand	67	67–67	…	…
**trnY**	N-strand	66	66–66	…	…
**COX1**	J-strand	1539	1539–1539	CGA	TAA
**trnL2**	J-strand	67	67–67	…	…
**COX2**	J-strand	685	685–685	ATG	T
**trnK**	J-strand	71	71–71	…	…
**trnD**	J-strand	68	68–68	…	…
**ATP8**	J-strand	162	162–162	ATT	TAA
**ATP6**	J-strand	681	681–681	ATG	TAA
**COX3**	J-strand	789	789–789	ATG	TAA
**trnG**	J-strand	67	67–67	…	…
**ND3**	J-strand	354	354–354	ATT	TAA
**trnR**	J-strand	64	64–64	…	…
**trnA**	J-strand	68	68–68	…	…
**trnN**	J-strand	67	67–67	…	…
**trnS1**	N-strand	67	67–67	…	…
**trnE**	J-strand	66	66–67	…	…
**trnF**	N-strand	67	67–67	…	…
**ND5**	N-strand	1743	1743–1743	GTG	TAA
**trnH**	N-strand	69	66–69	…	…
**ND4**	N-strand	1344	1344–1344	ATG	TAA
**ND4L**	N-strand	297	297–297	ATG	TAA
**trnT**	J-strand	65	65–67	…	…
**trnP**	N-strand	67	67–67	…	…
**ND6**	J-strand	522	522–522	ATT	TAA
**CYTB**	J-strand	1137	1137–1137	ATG	TAA
**trnS2**	J-strand	66	66–66	…	…
**ND1**	N-strand	951	951–951	TTG	TAA
**trnL1**	N-strand	68	68–68	…	…
**16S rRNA**	N-strand	1336	1336–1337	…	…
**trnV**	N-strand	72	72–72	…	…
**12S rRNA**	N-strand	797	797–797	…	…
**Control region**	J-strand	1767	948–2184	…	…

### Nucleotide Composition

The mitochondrial genomes of the Turkish *Ae. albopictus* isolates were strongly AT-rich, with an average A+T content of 79.93% and a corresponding GC content of 20.03%. Mean nucleotide frequencies across the complete mitogenomes were 40.03% A, 39.90% T, 11.88% C, and 8.19% G. Protein-coding genes accounted for the largest proportion of the mitogenome, representing approximately 67.4% of the total length (about 11,230 bp). Within the protein-coding gene partition, T was more abundant than A (44.56% vs. 32.83%), resulting in a negative AT skew (−0.15), whereas GC skew was slightly positive (0.04). The 2 ribosomal RNA genes comprised approximately 12.8% of the mitogenome (about 2,133 bp) and were also AT-rich (82.65%), with a distinctly positive GC skew (0.31), reflecting a higher proportion of G (11.45%) than C (6.06%). The 22 transfer RNA genes represented about 9.0% of the mitogenome (about 1,488 bp) and showed a more balanced distribution of A and T (AT skew = 0.0154), while remaining AT-rich overall (79.90%) and exhibiting a positive GC skew (0.13). Among all genome partitions, the control region showed the highest A + T content (92.62%), together with the lowest GC content (7.38%) and a strongly negative GC skew (−0.40), indicating a higher proportion of C (5.15%) than G (2.24%; Supplementary Table S2).

### Codon Usage Bias in Mitochondrial Protein-Coding Genes

**Fig. 3.**
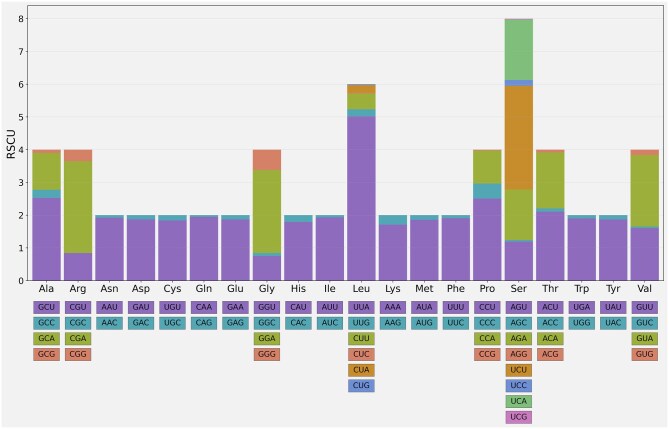
Relative synonymous codon usage (RSCU) profile of mitochondrial protein-coding genes in *Aedes albopictus*. Synonymous codons are grouped under each amino acid category, and leucine and serine codons are presented as single unified amino acid groups rather than separate subgroups.

Codon usage patterns across the 13 mitochondrial protein-coding genes were highly consistent among the 20 Turkish *Ae. albopictus* isolates, although minor quantitative differences were observed. Overall, codon usage showed a clear bias toward codons ending in A or U (Fig. 3), consistent with the strong A+T richness of the mitochondrial genomes. Among the most overrepresented codons, UUA encoding leucine showed the highest RSCU value, followed by UCU encoding serine. Relatively elevated RSCU values were also observed for CGA (arginine), GGA (glycine), CCU (proline), GCU (alanine), GUA (valine), and ACU (threonine). In contrast, codons ending in G or C were generally used less frequently; CGC encoding arginine was not detected, whereas AGG encoding serine showed a very low RSCU value. Across most synonymous codon families, A/U-ending codons were consistently favored over their G/C-ending alternatives. For example, UUA was strongly preferred among leucine codons, whereas UUU, AUU, CCU, ACU, and GUA were the most frequently used codons for phenylalanine, isoleucine, proline, threonine, and valine, respectively. Detailed codon counts and relative synonymous codon usage values are provided in Supplementary Table S3.

### Secondary Structures of Mitochondrial tRNAs and rRNAs

The 22 canonical mitochondrial transfer RNA genes were identified in all 20 Turkish *Ae. albopictus* mitogenomes, with predicted lengths ranging from 64 to 73 bp. Among these, trnR had the shortest predicted structure (64 bp), whereas trnV was the longest (72 bp). Predicted secondary structures were consistent with the compact organization typical of insect mitochondrial tRNAs, although minor variation was observed in stem length, loop size, and pairing patterns among genes. Distinct structural conformations were evident for the duplicated amino acid families, including trnL(UUR) and trnL(CUN), as well as trnS(AGN) and trnS(UCN). Overall, the predicted tRNA structures supported the accuracy of the gene annotations and the general architectural conservation of the *Ae. albopictus* mitogenomes (Supplementary Fig. S1).

Predicted secondary structures of the 2 mitochondrial ribosomal RNA genes are shown in Supplementary Fig. S2. Both rRNA genes exhibited the expected conserved structural organization of mosquito mitochondrial rRNAs, with no major differences among Turkish isolates. Together, these results indicate that the non-coding RNA components of the mitogenome are structurally conserved across the analyzed samples.

### Gene-Wise Mitochondrial Variation among Turkish Isolates

**Fig. 4.**
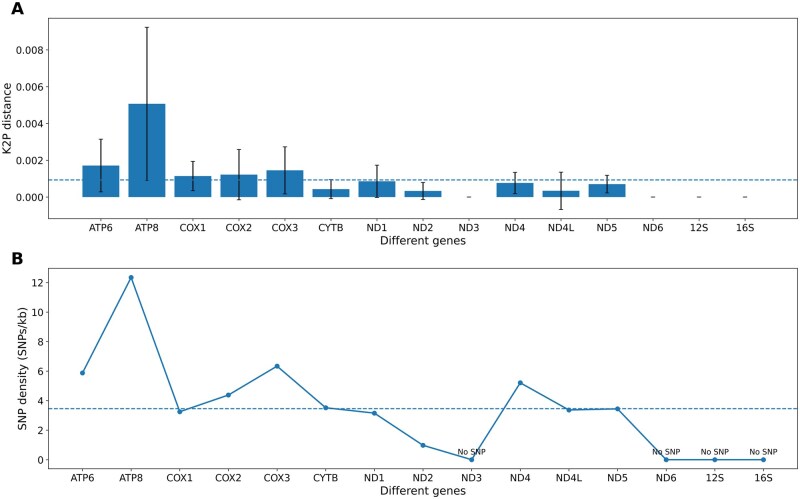
Gene-wise mitochondrial variation among 20 Turkish *Ae. albopictus* isolates: (A) mean Kimura 2-parameter (K2P) distances and (B) SNP density across 13 mitochondrial protein-coding genes and two ribosomal RNA genes.

Gene-wise mitochondrial variation across the 20 Turkish *Ae. albopictus* isolates is summarized in Fig. 4. Levels of sequence divergence were generally low, but varied among loci. The highest mean Kimura 2-parameter (K2P) distances were observed in ATP8 (0.0051 ± 0.0042), ATP6 (0.0017 ± 0.0014), and COX3 (0.0015 ± 0.0013), whereas no variation was detected in ND3, ND6, 12S rRNA, or 16S rRNA (Fig. 4A). Intermediate levels of divergence were observed in COX2, COX1, ND1, ND4, ND5, CYTB, ND4L, and ND2. Overall, these results indicate that mitochondrial variation among Turkish isolates was unevenly distributed across genes, with ATP8 showing markedly higher divergence than the remaining loci.

The distribution of single-nucleotide polymorphisms also varied among genes (Fig. 4B). ATP8 contained the highest SNP density (12.35 SNPs/kb), followed by COX3 (6.34 SNPs/kb) and ATP6 (5.87 SNPs/kb). Lower SNP densities were observed in ND4 (5.21 SNPs/kb), COX2 (4.38 SNPs/kb), CYTB (3.52 SNPs/kb), ND5 (3.44 SNPs/kb), ND4L (3.37 SNPs/kb), COX1 (3.25 SNPs/kb), ND1 (3.15 SNPs/kb), and ND2 (0.97 SNPs/kb), whereas no SNPs were detected in ND3, ND6, 12S rRNA, or 16S rRNA. Taken together, these results show that mitochondrial diversity in Turkish *Ae. albopictus* was concentrated in a limited number of loci, while most genes were highly conserved.

### Phylogenetic Relationships of Turkish Mitogenomes

**Fig. 5.**
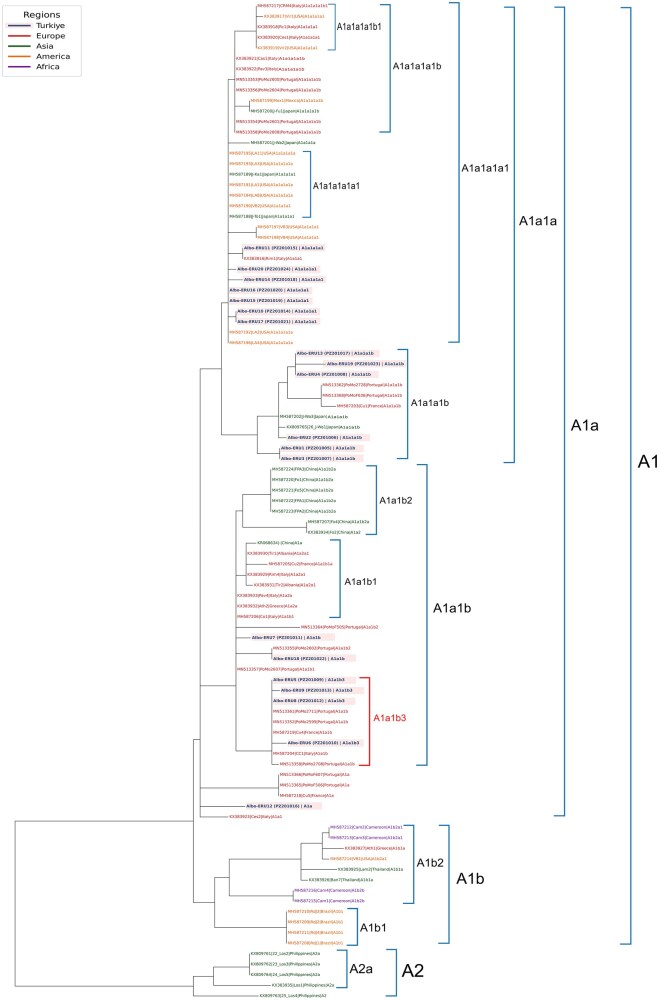
Maximum-likelihood phylogenetic tree of 95 *Ae. albopictus* mitogenomes inferred from concatenated sequences of 13 mitochondrial protein-coding genes and two ribosomal RNA genes. Turkish isolates are indicated by sample ID and locality, tip colors represent country of origin, and haplogroup assignments were made according to Battaglia et al. (2022).

Phylogenetic analysis of 95 *Ae. albopictus* mitogenomes based on concatenated sequences of 13 mitochondrial protein-coding genes and two ribosomal RNA genes showed that the Turkish samples did not form a single monophyletic group. Instead, they were distributed across several subclades within the broader mitochondrial diversity represented in the tree (Fig. 5). All Turkish isolates fell within the broad A1 lineage, and most were placed within the A1a complex. Overall, the Turkish samples were interspersed among reference mitogenomes from Europe, Asia, and North America, indicating that the populations analyzed here comprise multiple maternal lineages rather than a single country-specific mitochondrial lineage.

A major component of the Turkish dataset was assigned to the upper A1a1a sector of the tree. Within this broader assemblage, two distinct Turkish groupings were evident. The first included Albo-ERU10, Albo-ERU11, Albo-ERU14, Albo-ERU15, Albo-ERU16, Albo-ERU17, and Albo-ERU20, which were placed in the A1a1a1a1 lineage. The second included Albo-ERU1, Albo-ERU2, Albo-ERU3, Albo-ERU4, Albo-ERU13, and Albo-ERU19, which clustered within A1a1a1b. These 2 Turkish subgroups occurred in adjacent parts of the tree but remained phylogenetically distinct, indicating that even within the main A1a1a background, Turkish populations contain more than one maternal component.

A separate Turkish set was located within the broader A1a1b sector. Albo-ERU7 and Albo-ERU18 grouped within A1a1b, whereas Albo-ERU5, Albo-ERU6, Albo-ERU8, and Albo-ERU9 formed a distinct cluster labeled A1a1b3. In the final tree, this A1a1b3 cluster was separated from the adjacent A1a1b1 and A1a1b2 components and highlighted as an independent sublineage, suggesting an additional mitochondrial background among Turkish samples that is distinct from the main A1a1a-derived groupings.

One Turkish isolate, Albo-ERU12, was positioned separately within A1a, outside the main Turkish clusters assigned to A1a1a1a1, A1a1a1b, A1a1b, and A1a1b3. This isolated placement further supports the presence of marked mitogenomic heterogeneity among Turkish *Ae. albopictus* populations.

Taken together, the Turkish mitogenomes showed a mosaic phylogenetic distribution rather than a simple west-to-east geographic pattern (Fig. 1). Several Turkish isolates were closely intermingled with non-Turkish reference sequences, and no clear separation between Turkish and non-Turkish samples was observed. Most Turkish isolates were associated with subclades dominated by European reference mitogenomes, although some occurred in parts of the tree that also included Asian and North American sequences.

## Discussion

Mitochondrial genome analysis of *Ae. albopictus* populations from the Turkish Black Sea region showed conservation of the canonical mosquito mitochondrial gene repertoire and gene order, together with moderate variation in mitogenome length. This pattern is consistent with previous mitogenomic studies of *Ae. albopictus* and other mosquitoes (Battaglia et al. 2016, 2022, Zhang et al. 2016, Hao et al. 2017, Zé-Zé et al. 2020). The observed size variation was mainly attributable to the control region, a highly A+T-rich and structurally variable non-coding region in which tandem repeats, insertion/deletion events, and repeat copy-number differences commonly generate intraspecific length polymorphism (Zhang et al. 1995, Zhang and Hewitt 1997, Dueñas et al. 2006, Battaglia et al. 2016, 2022, Li and Liang 2018, Ji et al. 2019, Zé-Zé et al. 2020, Lin et al. 2021, Islam et al. 2025, Akintola and Hwang 2026). The absence of gene rearrangements in the Turkish mitogenomes indicates that the mitochondrial diversification detected here is associated primarily with sequence-level and control-region variation rather than large-scale structural reorganization, unlike rearrangements reported in some other mosquito mitogenomes (Lorenz et al. 2019, da Silva et al. 2025).

The phylogenetic results are broadly consistent with the global mitogenomic framework proposed by Battaglia et al. (2016, 2022), in which haplogroup A1, particularly the A1a complex, represents the dominant mitochondrial background associated with the recent worldwide expansion of *Ae. albopictus*. Turkish mitogenomes did not form a distinct country-specific lineage; instead, they were distributed across several branches within the A1/A1a radiation. This pattern indicates that *Ae. albopictus* populations from the Turkish Black Sea region are embedded within the broader intercontinental diversity of invasive *Ae. albopictus* rather than representing a deeply differentiated local lineage. At the same time, their placement in multiple sublineages shows that the Turkish populations analyzed here are not mitochondrially homogeneous and were unlikely to derive from a single maternal founder lineage.

Within the A1a background, Turkish samples were assigned to several closely related but distinct operational lineages, including A1a1a1a1, A1a1a1b, A1a1b, A1a1b3, and A1a. This distribution supports a scenario involving multiple maternal inputs, repeated introductions, secondary dispersal, or local reshuffling of already diversified invasive lineages. The A1a1b3 cluster should be interpreted cautiously because it was recovered in the present independently reconstructed phylogeny based on 13 mitochondrial protein-coding genes and two rRNA genes, rather than being a formally established haplogroup in the published Battaglia nomenclature. Therefore, the strongest agreement with Battaglia et al. (2016, 2022) is at the level of the major A1/A1a structure, whereas some fine-scale sublineage delimitations may differ because of differences in taxon sampling, gene matrix composition, and analytical strategy. Overall, the Turkish data support the Battaglia model of a diversified invasive A1a radiation shaped by founder events, while also indicating that *Ae. albopictus* populations in Türkiye represent a locally complex combination of maternal lineages rather than a single Battaglia-defined sub-branch.

The heterogeneous and non-monophyletic pattern observed among the Turkish samples is consistent with the broader invasion literature on *Ae. albopictus*. The worldwide expansion of this species has been associated with ecological plasticity, passive transport, and repeated establishment in anthropized environments, rather than with a simple radial spread from a single source (Medlock et al. 2012, Bonizzoni et al. 2013, Corley et al. 2025). This interpretation is also supported by surveillance studies from Türkiye, which documented the first detection of *Ae. albopictus* in Thrace, its subsequent expansion along the Black Sea region, its persistence and high local densities in the eastern Black Sea area, and its continuing range expansion over time (Oter et al. 2013, Akıner et al. 2016, 2018, 2019, Demirci et al. 2021). Our mitogenomic data add further resolution to this epidemiological and ecological picture by showing that the geographical expansion of *Ae. albopictus* in Türkiye was not associated with a single mitochondrial origin but with several distinct maternal lineages, in some cases occurring within the same province.

The mosaic-like organization of Turkish mitogenomes is also compatible with population genetic and genomic studies showing that the global invasion of *Ae. albopictus* has been shaped by chaotic human-mediated dispersal, multiple founding events, regional connectivity, admixture, and local establishment rather than by a simple stepwise diffusion process (Kotsakiozi et al. 2017, Manni et al. 2017, Sherpa et al. 2018, 2019, Pichler et al. 2019, Vavassori et al. 2022, Corley et al. 2025). The absence of a clear west-to-east gradient in our Turkish dataset, together with the coexistence of several mitochondrial backgrounds across sampled provinces, fits this repeated and composite invasion model. Similar mitogenomic patterns have been reported in Portugal and in broader global datasets, where adventive populations were associated with multiple introductions, secondary dispersal, and widespread A1/A1a-derived lineages (Zé-Zé et al. 2020, Battaglia et al. 2022). Nevertheless, exact dispersal routes should be interpreted cautiously. As emphasized by Battaglia et al. (2016, 2022), mitogenomic phylogeny can suggest phylogeographic affinities, but close placement with Japanese, European, or North American references does not by itself demonstrate direct introduction from those regions into Türkiye. These relationships are better regarded as phylogenetic neighborhoods or putative invasion scenarios, potentially mediated by bridgehead populations and secondary redistribution within the established global invasive range.

From an applied perspective, the recovery of multiple maternal lineages in Türkiye has implications for biosecurity and vector management. Continued introductions of *Ae. albopictus* may facilitate the influx not only of new mitochondrial backgrounds but also of adaptive traits associated with invasion success, including insecticide resistance profiles documented in some extra-regional populations. Although *Ae. albopictus* is already established in Türkiye, strengthening surveillance at likely entry points, such as ports, commercial transport routes, and urbanized coastal corridors, may help reduce further introductions and secondary spread. Mitogenomic monitoring could complement routine entomological surveillance by identifying newly introduced maternal lineages and tracking their spatial expansion.

## Supplementary Material

tjag109_Supplementary_Data

## Data Availability

The newly generated mitochondrial genome sequences were deposited in GenBank under accession numbers PZ201005–PZ201024. The custom Python pipeline used for sequence retrieval, matrix construction, and phylogenetic data processing is available from the corresponding author upon reasonable request.
