# Supplementary material for: Mitogenomic diversity and phylogenetic characterization of Aedes albopictus (Diptera: Culicidae) populations from the Black Sea region of Türkiye
Source: J Med Entomol. 2026 Jul 3;63(4):tjag109. doi: 10.1093/jme/tjag109 (PMC13332435; doi:10.1093/jme/tjag109)
Supplement: tjag109_Supplementary_Data [file tjag109_supplementary_data.zip › Supplementary_Table_S1.docx]

**Supplementary Table S1.** Information about the samples.

| **New sample ID** | **Location** | **Raw sequences** | **Trimmed, quality-filtered and assembled sequences** | **Coverage mean (X)** | **Mitogenome length (bp)** |
| --- | --- | --- | --- | --- | --- |
| Albo-ERU1 | Giresun | 38,392,936 | 28,804,371 | 622 | 15,843 |
| Albo-ERU2 | Ordu | 66,464,594 | 47,108,629 | 121 | 15,861 |
| Albo-ERU3 | Sinop | 47,424,490 | 34,685,648 | 331 | 16,436 |
| Albo-ERU4 | Samsun | 59,012,904 | 44,308,600 | 569 | 16,864 |
| Albo-ERU5 | Istanbul | 63,915,904 | 47,730,095 | 587 | 16,571 |
| Albo-ERU6 | Istanbul | 95,629,430 | 71,507,905 | 1,834 | 17,076 |
| Albo-ERU7 | Artvin | 206,905,804 | 153,566,451 | 5,183 | 17,014 |
| Albo-ERU8 | Cide | 55,451,226 | 40,573,017 | 490 | 16,654 |
| Albo-ERU9 | Kırklareli | 85,371,522 | 64,537,831 | 290 | 16,654 |
| Albo-ERU10 | Artvin | 193,042,334 | 144,560,156 | 8,335 | 16,661 |
| Albo-ERU11 | Ordu | 42,165,722 | 30,134,415 | 75 | 16,655 |
| Albo-ERU12 | Kırklareli | 74,442,288 | 55,424,097 | 111 | 16,662 |
| Albo-ERU13 | Samsun | 60,843,974 | 45,307,061 | 169 | 16,661 |
| Albo-ERU14 | Rize | 189,349,502 | 131,534,038 | 272 | 16,662 |
| Albo-ERU15 | Trabzon | 45,132,160 | 33,110,425 | 79 | 16,662 |
| Albo-ERU16 | Rize | 263,311,242 | 196,176,907 | 1,021 | 16,662 |
| Albo-ERU17 | Giresun | 28,544,440 | 20,963,101 | 37.5 | 16,648 |
| Albo-ERU18 | Bartın | 69,926,268 | 51,384,600 | 124 | 16,661 |
| Albo-ERU19 | Sinop | 42,165,276 | 31,290,985 | 37 | 16,664 |
| Albo-ERU20 | Trabzon | 346,172,630 | 260,728,709 | 1,572 | 16,667 |
