# Supplementary material for: Mitogenomic diversity and phylogenetic characterization of Aedes albopictus (Diptera: Culicidae) populations from the Black Sea region of Türkiye
Source: J Med Entomol. 2026 Jul 3;63(4):tjag109. doi: 10.1093/jme/tjag109 (PMC13332435; doi:10.1093/jme/tjag109)
Supplement: tjag109_Supplementary_Data [file tjag109_supplementary_data.zip › Supplementary_Table_S2.docx]

**Supplementary Table S2.** Regional nucleotide composition of the complete mitochondrial genomes of 20 Turkish *Ae. albopictus* isolates.

| **Region** | **Size (bp)** | **A (%)** | **T (%)** | **G (%)** | **C (%)** | **A+T (%)** | **G+C (%)** | **AT-skew** | **GC-skew** |
| --- | --- | --- | --- | --- | --- | --- | --- | --- | --- |
| Whole mitogenome | 16611.90 ± 295.41 | 40.03 ± 0.08 | 39.90 ± 0.21 | 8.19 ± 0.16 | 11.88 ± 0.14 | 79.93 ± 0.30 | 20.07 ± 0.30 | 0.0017 ± 0.0018 | -0.1837 ± 0.0040 |
| Total PCGs | 11230 ± 0.00 | 32.83 ± 0.02 | 44.56 ± 0.01 | 11.76 ± 0.02 | 10.85 ± 0.01 | 77.39 ± 0.02 | 22.61 ± 0.02 | -0.1515 ± 0.0004 | 0.0403 ± 0.0011 |
| Total tRNAs | 1487.60 ± 0.88 | 40.57 ± 0.04 | 39.34 ± 0.05 | 11.37 ± 0.05 | 8.73 ± 0.03 | 79.90 ± 0.06 | 20.10 ± 0.06 | 0.0154 ± 0.0008 | 0.1313 ± 0.0027 |
| 16S rRNA | 1336.05 ± 0.22 | 38.92 ± 0.01 | 44.16 ± 0.01 | 11.23 ± 0.00 | 5.69 ± 0.00 | 83.08 ± 0.00 | 16.92 ± 0.00 | -0.0631 ± 0.0002 | 0.3274 ± 0.0000 |
| 12S rRNA | 797 ± 0.00 | 39.90 ± 0.00 | 42.03 ± 0.00 | 11.67 ± 0.00 | 6.40 ± 0.00 | 81.93 ± 0.00 | 18.07 ± 0.00 | -0.0260 ± 0.0000 | 0.2917 ± 0.0000 |
| Control region | 1717.55 ± 295.89 | 43.91 ± 0.43 | 48.70 ± 0.92 | 2.24 ± 0.92 | 5.15 ± 0.45 | 92.62 ± 1.18 | 7.38 ± 1.18 | -0.0516 ± 0.0086 | -0.4075 ± 0.1039 |
| ND2 | 1026 ± 0.00 | 36.73 ± 0.04 | 46.30 ± 0.00 | 7.72 ± 0.04 | 9.26 ± 0.00 | 83.02 ± 0.04 | 16.98 ± 0.04 | -0.1153 ± 0.0005 | -0.0907 ± 0.0026 |
| COX1 | 1539 ± 0.00 | 29.90 ± 0.05 | 40.05 ± 0.03 | 14.35 ± 0.05 | 15.70 ± 0.03 | 69.95 ± 0.05 | 30.05 ± 0.05 | -0.1451 ± 0.0010 | -0.0449 ± 0.0022 |
| COX2 | 685 ± 0.00 | 35.61 ± 0.03 | 41.45 ± 0.03 | 9.79 ± 0.03 | 13.15 ± 0.03 | 77.07 ± 0.04 | 22.93 ± 0.04 | -0.0758 ± 0.0006 | -0.1464 ± 0.0021 |
| ATP8 | 162 ± 0.00 | 39.91 ± 0.30 | 45.15 ± 0.46 | 4.94 ± 0.00 | 10.00 ± 0.25 | 85.06 ± 0.25 | 14.94 ± 0.25 | -0.0617 ± 0.0085 | -0.3387 ± 0.0109 |
| ATP6 | 681 ± 0.00 | 31.53 ± 0.13 | 42.28 ± 0.03 | 10.32 ± 0.13 | 15.87 ± 0.03 | 73.81 ± 0.14 | 26.19 ± 0.14 | -0.1457 ± 0.0019 | -0.2117 ± 0.0058 |
| COX3 | 789 ± 0.00 | 30.41 ± 0.06 | 41.83 ± 0.00 | 13.06 ± 0.06 | 14.70 ± 0.00 | 72.24 ± 0.06 | 27.76 ± 0.06 | -0.1580 ± 0.0010 | -0.0591 ± 0.0025 |
| ND3 | 354 ± 0.00 | 36.44 ± 0.00 | 45.20 ± 0.00 | 8.19 ± 0.00 | 10.17 ± 0.00 | 81.64 ± 0.00 | 18.36 ± 0.00 | -0.1073 ± 0.0000 | -0.1077 ± 0.0000 |
| ND5 | 1743 ± 0.00 | 33.33 ± 0.02 | 46.21 ± 0.03 | 12.85 ± 0.02 | 7.60 ± 0.03 | 79.55 ± 0.04 | 20.45 ± 0.04 | -0.1619 ± 0.0004 | 0.2567 ± 0.0021 |
| ND4 | 1344 ± 0.00 | 32.90 ± 0.05 | 46.87 ± 0.02 | 12.94 ± 0.05 | 7.30 ± 0.02 | 79.77 ± 0.05 | 20.23 ± 0.05 | -0.1751 ± 0.0008 | 0.2785 ± 0.0025 |
| ND4L | 297 ± 0.00 | 31.30 ± 0.08 | 51.52 ± 0.00 | 12.14 ± 0.08 | 5.05 ± 0.00 | 82.81 ± 0.08 | 17.19 ± 0.08 | -0.2442 ± 0.0011 | 0.4123 ± 0.0025 |
| ND6 | 522 ± 0.00 | 38.51 ± 0.00 | 45.59 ± 0.00 | 5.94 ± 0.00 | 9.96 ± 0.00 | 84.10 ± 0.00 | 15.90 ± 0.00 | -0.0843 ± 0.0000 | -0.2530 ± 0.0000 |
| CYTB | 1137 ± 0.00 | 31.84 ± 0.03 | 43.18 ± 0.03 | 12.31 ± 0.03 | 12.66 ± 0.03 | 75.03 ± 0.04 | 24.97 ± 0.04 | -0.1512 ± 0.0006 | -0.0143 ± 0.0018 |
| ND1 | 951 ± 0.00 | 29.32 ± 0.04 | 48.41 ± 0.05 | 14.53 ± 0.04 | 7.74 ± 0.05 | 77.73 ± 0.04 | 22.27 ± 0.04 | -0.2455 ± 0.0010 | 0.3045 ± 0.0038 |
