# Supplementary material for: Mitogenomic diversity and phylogenetic characterization of Aedes albopictus (Diptera: Culicidae) populations from the Black Sea region of Türkiye
Source: J Med Entomol. 2026 Jul 3;63(4):tjag109. doi: 10.1093/jme/tjag109 (PMC13332435; doi:10.1093/jme/tjag109)
Supplement: tjag109_Supplementary_Data [file tjag109_supplementary_data.zip › Supplementary_Table_S3.docx]

**Supplementary Table S3.** Codon counts and relative synonymous codon usage (RSCU) of the 13 mitochondrial protein-coding genes in 20 Turkish *Ae. albopictus* isolates. Values are presented as mean ± SD and observed range across isolates.

| **Amino acid** | **Codon** | **Count (mean ± SD)** | **Observed count range** | **RSCU (mean ± SD)** | **Observed RSCU range** |
| --- | --- | --- | --- | --- | --- |
| Ala | GCU | 100.00 ± 0.79 | 99–101 | 2.511 ± 0.017 | 2.475–2.541 |
| Ala | GCC | 11.00 ± 0.79 | 10–12 | 0.276 ± 0.020 | 0.250–0.302 |
| Ala | GCA | 45.05 ± 0.22 | 45–46 | 1.131 ± 0.005 | 1.125–1.150 |
| Ala | GCG | 3.25 ± 0.44 | 3–4 | 0.082 ± 0.011 | 0.075–0.100 |
| Arg | CGU | 12.00 ± 0.00 | 12–12 | 0.842 ± 0.000 | 0.842–0.842 |
| Arg | CGC | 0.00 ± 0.00 | 0–0 | 0.000 ± 0.000 | 0.000–0.000 |
| Arg | CGA | 40.00 ± 0.00 | 40–40 | 2.807 ± 0.000 | 2.807–2.807 |
| Arg | CGG | 5.00 ± 0.00 | 5–5 | 0.351 ± 0.000 | 0.351–0.351 |
| Asn | AAU | 211.75 ± 0.64 | 211–213 | 1.918 ± 0.000 | 1.918–1.919 |
| Asn | AAC | 9.00 ± 0.00 | 9–9 | 0.082 ± 0.000 | 0.081–0.082 |
| Asp | GAU | 58.80 ± 0.41 | 58–59 | 1.870 ± 0.010 | 1.841–1.873 |
| Asp | GAC | 4.10 ± 0.31 | 4–5 | 0.130 ± 0.010 | 0.127–0.159 |
| Cys | UGU | 34.00 ± 0.00 | 34–34 | 1.838 ± 0.000 | 1.838–1.838 |
| Cys | UGC | 3.00 ± 0.00 | 3–3 | 0.162 ± 0.000 | 0.162–0.162 |
| Gln | CAA | 73.85 ± 0.37 | 73–74 | 1.943 ± 0.010 | 1.921–1.947 |
| Gln | CAG | 2.15 ± 0.37 | 2–3 | 0.057 ± 0.010 | 0.053–0.079 |
| Glu | GAA | 71.00 ± 0.00 | 71–71 | 1.868 ± 0.000 | 1.868–1.868 |
| Glu | GAG | 5.00 ± 0.00 | 5–5 | 0.132 ± 0.000 | 0.132–0.132 |
| Gly | GGU | 39.85 ± 0.49 | 38–40 | 0.748 ± 0.009 | 0.714–0.751 |
| Gly | GGC | 5.15 ± 0.49 | 5–7 | 0.097 ± 0.009 | 0.094–0.131 |
| Gly | GGA | 134.75 ± 0.44 | 134–135 | 2.531 ± 0.008 | 2.516–2.535 |
| Gly | GGG | 33.25 ± 0.44 | 33–34 | 0.624 ± 0.008 | 0.620–0.638 |
| His | CAU | 69.00 ± 0.00 | 69–69 | 1.792 ± 0.000 | 1.792–1.792 |
| His | CAC | 8.00 ± 0.00 | 8–8 | 0.208 ± 0.000 | 0.208–0.208 |
| Ile | AUU | 358.35 ± 0.81 | 357–359 | 1.929 ± 0.002 | 1.925–1.930 |
| Ile | AUC | 13.25 ± 0.44 | 13–14 | 0.071 ± 0.002 | 0.070–0.075 |
| Leu | CUU | 47.00 ± 0.00 | 47–47 | 0.481 ± 0.000 | 0.480–0.481 |
| Leu | CUC | 2.00 ± 0.00 | 2–2 | 0.020 ± 0.000 | 0.020–0.020 |
| Leu | CUA | 23.00 ± 0.32 | 22–24 | 0.235 ± 0.003 | 0.225–0.246 |
| Leu | CUG | 3.20 ± 0.41 | 3–4 | 0.033 ± 0.004 | 0.031–0.041 |
| Leu | UUA | 490.35 ± 0.75 | 490–493 | 5.020 ± 0.006 | 5.017–5.039 |
| Leu | UUG | 20.50 ± 0.89 | 19–21 | 0.210 ± 0.009 | 0.194–0.215 |
| Lys | AAA | 83.20 ± 0.41 | 83–84 | 1.715 ± 0.008 | 1.711–1.732 |
| Lys | AAG | 13.80 ± 0.41 | 13–14 | 0.285 ± 0.008 | 0.268–0.289 |
| Met | AUA | 241.85 ± 1.09 | 241–244 | 1.858 ± 0.005 | 1.854–1.869 |
| Met | AUG | 18.50 ± 0.61 | 17–19 | 0.142 ± 0.005 | 0.131–0.146 |
| Phe | UUU | 348.00 ± 0.00 | 348–348 | 1.907 ± 0.000 | 1.907–1.907 |
| Phe | UUC | 17.00 ± 0.00 | 17–17 | 0.093 ± 0.000 | 0.093–0.093 |
| Pro | CCU | 82.75 ± 0.44 | 82–83 | 2.527 ± 0.014 | 2.504–2.534 |
| Pro | CCC | 14.25 ± 0.44 | 14–15 | 0.435 ± 0.014 | 0.427–0.458 |
| Pro | CCA | 33.00 ± 0.00 | 33–33 | 1.008 ± 0.000 | 1.008–1.008 |
| Pro | CCG | 1.00 ± 0.00 | 1–1 | 0.031 ± 0.000 | 0.031–0.031 |
| Ser | AGU | 47.00 ± 0.00 | 47–47 | 1.190 ± 0.001 | 1.190–1.194 |
| Ser | AGC | 2.00 ± 0.00 | 2–2 | 0.051 ± 0.000 | 0.051–0.051 |
| Ser | AGA | 61.00 ± 0.00 | 61–61 | 1.545 ± 0.001 | 1.544–1.549 |
| Ser | AGG | 0.00 ± 0.00 | 0–0 | 0.000 ± 0.000 | 0.000–0.000 |
| Ser | UCU | 125.00 ± 0.00 | 125–125 | 3.165 ± 0.002 | 3.165–3.175 |
| Ser | UCC | 7.00 ± 0.00 | 7–7 | 0.177 ± 0.000 | 0.177–0.178 |
| Ser | UCA | 73.00 ± 0.32 | 72–74 | 1.848 ± 0.007 | 1.829–1.873 |
| Ser | UCG | 0.95 ± 0.22 | 0–1 | 0.024 ± 0.006 | 0.000–0.025 |
| Thr | ACU | 100.95 ± 0.22 | 100–101 | 2.104 ± 0.006 | 2.083–2.115 |
| Thr | ACC | 5.05 ± 0.22 | 5–6 | 0.105 ± 0.005 | 0.104–0.125 |
| Thr | ACA | 81.00 ± 0.46 | 80–82 | 1.688 ± 0.008 | 1.675–1.708 |
| Thr | ACG | 4.90 ± 0.31 | 4–5 | 0.102 ± 0.006 | 0.083–0.105 |
| Trp | UGA | 94.95 ± 0.22 | 94–95 | 1.899 ± 0.004 | 1.880–1.900 |
| Trp | UGG | 5.05 ± 0.22 | 5–6 | 0.101 ± 0.004 | 0.100–0.120 |
| Tyr | UAU | 154.35 ± 0.49 | 154–155 | 1.867 ± 0.000 | 1.867–1.867 |
| Tyr | UAC | 11.00 ± 0.00 | 11–11 | 0.133 ± 0.000 | 0.133–0.133 |
| Val | GUU | 67.40 ± 0.50 | 67–68 | 1.606 ± 0.011 | 1.595–1.619 |
| Val | GUC | 2.00 ± 0.00 | 2–2 | 0.048 ± 0.000 | 0.047–0.048 |
| Val | GUA | 90.60 ± 0.75 | 88–91 | 2.159 ± 0.015 | 2.120–2.180 |
| Val | GUG | 7.85 ± 0.59 | 7–9 | 0.187 ± 0.014 | 0.167–0.217 |
