# Supplementary figures and images for: Mitogenomic diversity and phylogenetic characterization of Aedes albopictus (Diptera: Culicidae) populations from the Black Sea region of Türkiye
Source: J Med Entomol. 2026 Jul 3;63(4):tjag109. doi: 10.1093/jme/tjag109 (PMC13332435; doi:10.1093/jme/tjag109)

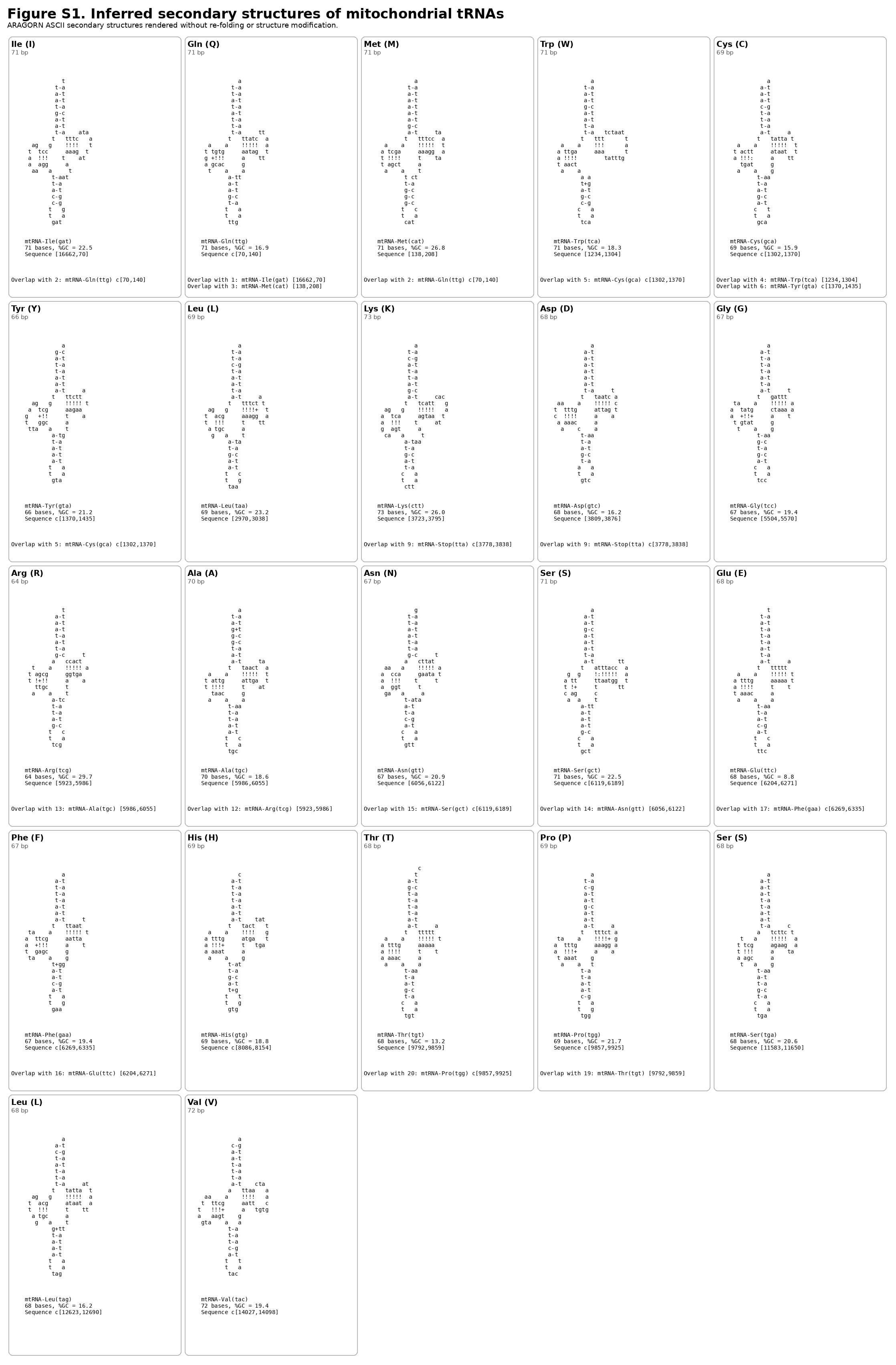

Supplement: tjag109_Supplementary_Data [file tjag109_supplementary_data.zip › Supplementary Figure S1.png]

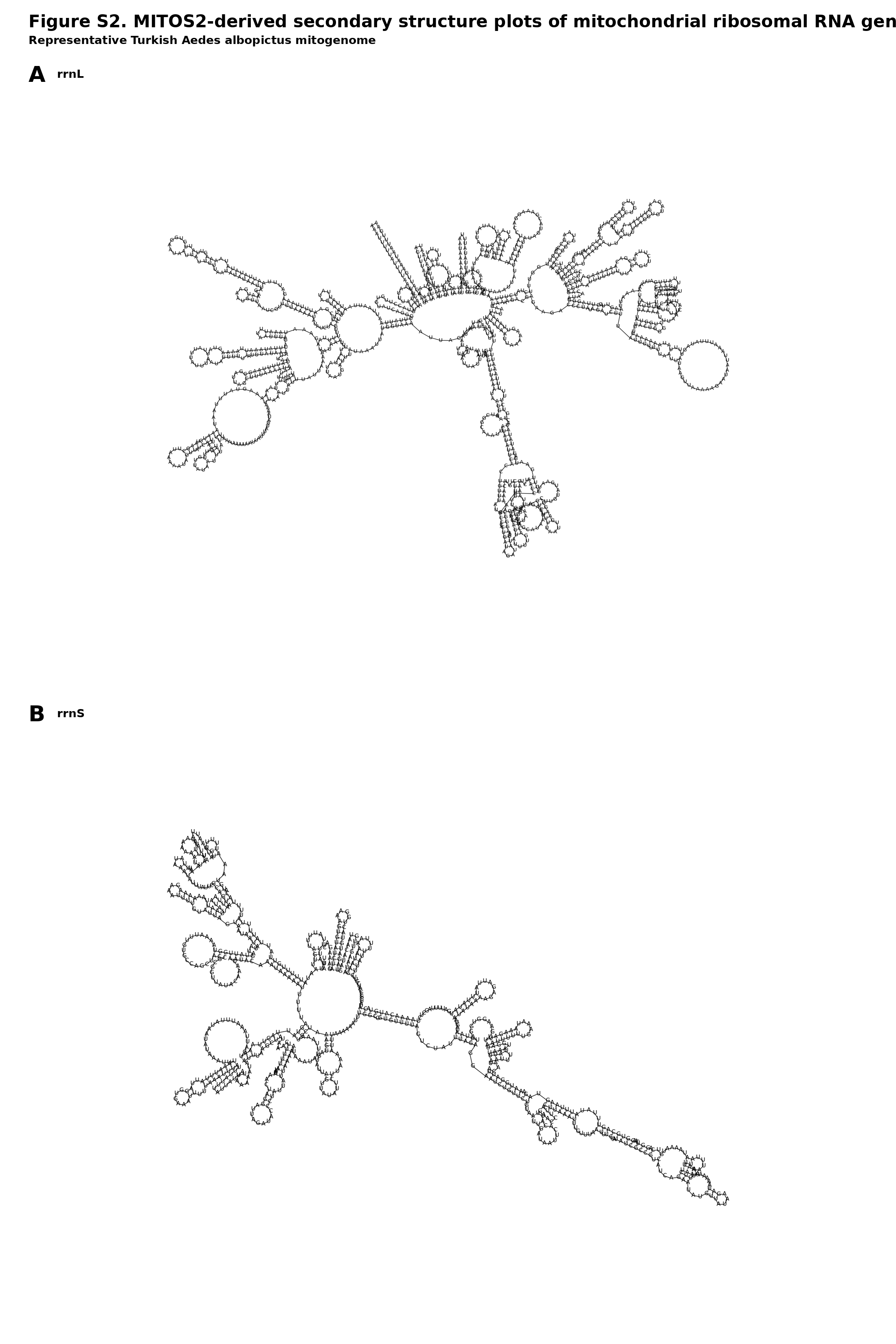

Supplement: tjag109_Supplementary_Data [file tjag109_supplementary_data.zip › Supplementary Figure S2.png]
